# A Case of Right Hepatic Vein Thrombus with Spontaneous Peripheral Shunt Successfully Treated via Left Trisectionectomy with Bile Duct Resection

**DOI:** 10.70352/scrj.cr.25-0332

**Published:** 2025-11-05

**Authors:** Takahiro Shoda, Kenichiro Araki, Norihiro Ishi, Ryosuke Fukushima, Takayuki Okuyama, Takaomi Seki, Kouki Hoshino, Kei Hagiwara, Shunsuke Kawai, Mariko Tsukagoshi, Takamichi Igarashi, Norio Kubo, Ken Shirabe

**Affiliations:** Division of Hepatobiliary and Pancreatic Surgery, Department of General Surgical Science, Gunma University Graduate School of Medicine, Maebashi, Gunma, Japan

**Keywords:** intraductal papillary neoplasm of bile duct, left hepatic trisectionectomy, right hepatic vein thrombosis, portal vein embolization, venous shunt

## Abstract

**INTRODUCTION:**

Left trisectionectomy with bile duct resection is a high-risk procedure that requires thorough preoperative evaluation to prevent postoperative liver failure. In addition, there are a few reports of highly invasive hepatectomy in cases where hepatic vein thrombosis is present preoperatively.

**CASE PRESENTATION:**

In a 58-year-old man, papillary epithelium was detected in the left hepatic duct during a bile duct biopsy, and the anterior segment of Glisson's capsule was compressed by the cystic components. Left trisectionectomy with bile duct resection was planned, based on a diagnosis of intraductal papillary neoplasm of the bile duct (IPNB). In our department, portal vein embolization (PVE) is essential when considering left trisectionectomy with bile duct resection, so surgery was scheduled after PVE. However, CT after PVE showed thrombus formation in the right hepatic vein (RHV), which persisted despite the initiation of anticoagulant therapy. Owing to the absence of a major drainage vein and the risk of postoperative liver failure, the patient was treated with gemcitabine + cisplatin + S-1 therapy. CT after chemotherapy still showed RHV thrombosis, along with newly developed peripheral venous shunt formation between the obliterated RHV branches. After 6 months, the same findings were observed, and as the tumor had shrunk, the case was deemed resectable. Left trisectionectomy with bile duct resection was performed. Pathological diagnosis confirmed IPNB (pTisN0M0, pStage 0 according to the Union for International Cancer Control, 8th edition). Following adjuvant chemotherapy, the patient developed pulmonary metastases, which were surgically resected. As of 38 months post-hepatectomy, the patient remains cancer-free.

**CONCLUSIONS:**

We encountered a case in which left trisectionectomy with bile duct resection was possible owing to the formation of an RHV shunt, enabling resection of both the primary and recurrent lesions. Continuous imaging is essential for the dynamic assessment of resectability.

## Abbreviations

ERCP
: endoscopic retrograde cholangiopancreatography
fFRLV
: functional future remnant liver volume
FLR
: future liver remnant
GCS
: gemcitabine/cisplatin/S-1
HVE
: hepatic vein embolization
ICG-K
: indocyanine green plasma clearance rate
ICG-Krem
: composite index calculated by multiplying ICG-K by the future remnant liver volume (rem)
IPNB
: intraductal papillary neoplasm of the bile duct
IRHV
: inferior right hepatic vein
IVC
: inferior vena cava
MHV
: middle hepatic vein
PVE
: portal vein embolization
RHV
: right hepatic vein

## INTRODUCTION

Left trisectionectomy with bile duct resection is a high-risk procedure with a reported 90-day mortality rate of 10.3% in hepatobiliary advanced skills training centers.^1)^ Detailed preoperative assessment is essential to prevent postoperative liver failure due to impaired hepatic blood flow and inadequate bile excretion. Few cases of left trisectionectomy with bile duct resection involving hepatic vein reconstruction have been reported. Nagino et al. described a case in which RHV HVE was performed preoperatively to enhance blood flow through the IRHV, followed by left trisectionectomy with bile duct resection preserving the IRHV.^2)^ Herein, we report a case of IPNB in the hilar region that was initially deemed unresectable owing to RHV thrombus. However, a hepatic vein shunt formed during chemotherapy, enabling successful left trisectionectomy with bile duct resection.

## CASE PRESENTATION

**Fig. 1:**
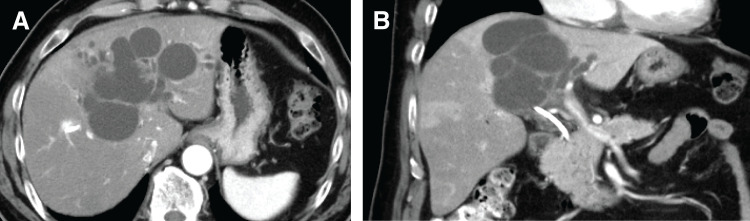
Enhanced CT and ERCP of the case (on admission to our hospital). (**A**, **B**) Enhanced CT showing a lesion protruding from the medial bile duct into the left hepatic duct, along with a dilated intrahepatic bile duct. ERCP, endoscopic retrograde cholangiopancreatography

A 58-year-old man visited his previous doctor with the main complaint of jaundice. He was referred to our department because cholangiocarcinoma in the hilar region was suspected based on CT findings. At our hospital, CT showed cystic dilatation of the bile ducts, mainly in the left lobe (**Fig. 1A** and **1B**). Furthermore, ERCP and mapping biopsy confirmed a papillary neoplasm originating from the left hepatic duct, which was suspected to be IPNB. Although there were no tumor components in the anterior/posterior sector bile ducts, the cystic components caused stenosis of the root of the anterior bile duct and compression of the anterior Glisson’s capsule. Therefore, left trisectionectomy with bile duct resection was planned. Child–Pugh and liver damage were both Grade A, and the ICG-Krem value was 0.052, indicating sufficient liver function for major hepatectomy. ICG-Krem is a composite index calculated by multiplying the ICG-K by the future remnant liver volume (rem), and is commonly used in Japan to estimate the functional capacity of the remnant liver.^3)^

**Fig. 2:**
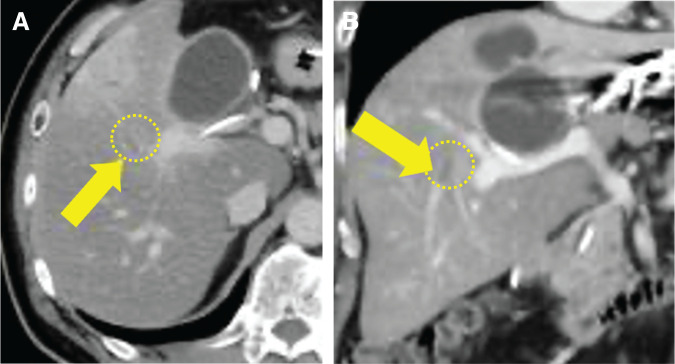
Enhanced CT after thrombus formation. (**A**, **B**) The arrows indicate the RHV, and the area enclosed by the broken line represents a blood clot. RHV, right hepatic vein

The FLR was 695 mL (39.2%), and the fFRLV, which is the functional future remnant liver volume assessed using MRI, was 723 mL/m^2^, satisfying our resection criteria (fFRLV ≥615 mL/m^2^).^2,4)^ As PVE is mandatory in our department before left trisectionectomy with bile duct resection, left PVE was performed accordingly. However, post-PVE CT showed a thrombus in the RHV, prompting the initiation of anticoagulation (**Fig. 2A** and **2B**). The thrombus persisted, raising suspicion of a tumor thrombus. There were no obvious collateral drainage vessels besides the RHV.

At this point, the ICG-Krem had increased to 0.083, and the FLR was 709 mL (40.03%). Impaired blood flow was observed in the peripheral RHV distal to the thrombus, and the congested liver volume was 299 mL (42% of the posterior segment). The adjusted fFRLV was 620 mL/m^2^, which barely met our resection criteria (fFRLV ≥615 mL/m^2^). However, there was still a risk of thrombus progression before or after surgery, and in addition, there was no drainage vein in the remaining liver, raising concerns about severe congestion.

**Fig. 3:**
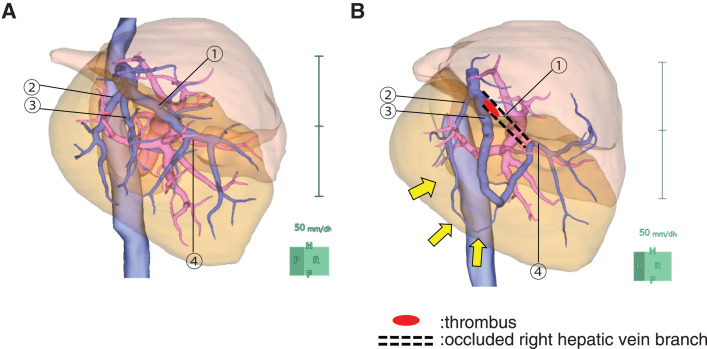
Volumetry before and after shunt formation. (**A**) Before shunt formation and (**B**) after shunt formation. A thrombus formed at the root of the RHV (①), while an intravenous shunt developed between the periphery of V7 (②), the periphery of another V7 (③), and the periphery of the main trunk of the RHV (④). The yellow arrows indicate the shunt. RHV, right hepatic vein

**Fig. 4:**
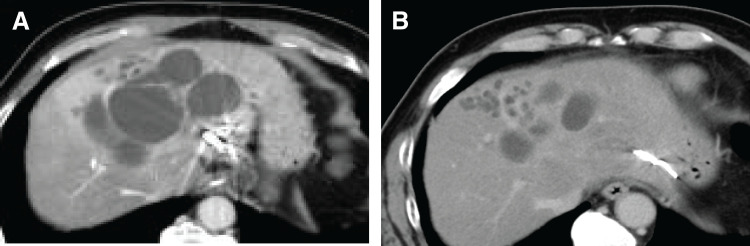
Enhanced CT after initiating GCS. (**A**) Taken 3 months later and (**B**) taken 6 months later. The tumor size decreased, and the remnant liver enlarged. GCS, gemcitabine/cisplatin/S-1

Balancing the risk of hepatic failure and the tumor’s curative potential, chemotherapy was initiated. The chosen regimen was GCS, in accordance with the Japanese clinical practice guidelines for biliary tract cancer. CT performed 3 months after initiating GCS showed persistent RHV thrombosis and the formation of a shunt at the obliterated RHV site, suggesting it developed during chemotherapy. At 6 months, CT still showed a residual shunt (**Fig. 3A** and **3B**), reduced tumor diameter, and increased FLR (**Fig. 4A** and **4B**). Throughout chemotherapy, the patient remained stable without jaundice or cholangitis. After confirming the growth of FLR (695→1232 mL), fFRLV (740 mL/m^2^), and ICG-Krem value (0.059), surgery was scheduled (**Table 1**). At the time of surgery, both the Child–Pugh classification and liver function impairment were grade A.

**Table 1:** Comparison of the remnant liver after left trisectionectomy

Evaluation period	Total liver volume (mL)	FLR	fFRLV (mL/m^2^)	ICG-K	ICG-Krem
Before PVE	1774	695 mL (39.18%)	723	0.132	0.052
After PVE	1771	709 mL (40.03%)	620	0.207	0.083
After shunt formation	1998	1232 mL (61.66%)	740	0.095	0.059

Liver enlargement ratio (after shunt formation/before PVE): 1.773.

fFRLV, functional future remnant liver volume; FLR, future liver remnant; ICG-K, idocyanine green plasma clearance rate; ICG-Krem, composite index calculated by multiplying ICG-K by the future remnant liver volume (rem); PVE, portal vein embolization

**Fig. 5:**
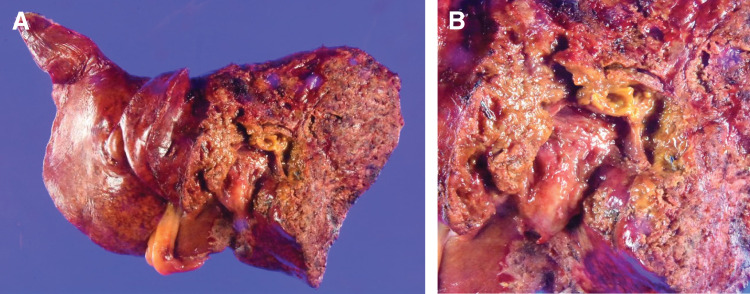
Pathological findings. (**A**) Gross appearance of the surgical resection specimen and (**B**) magnified view of the surgical margin Eg, s0, n0, p0, sm(−), ch, f1–2, pTisN0M0, pStage0.

Left trisectionectomy with bile duct resection (H123458-B)^5)^ was performed 6 months later than initially planned. Intraoperatively, Doppler ultrasound confirmed antegrade blood flow from the shunt to the central RHV. The operation lasted 12 h and 45 min, with 1318 mL of blood loss. Transfusion included 280 mL of red blood cells and 480 mL of fresh frozen plasma. Histopathology confirmed IPNB with intraepithelial carcinoma, negative surgical margins, and no lymph node metastasis: pTisN0M0, pStage 0 (Union for International Cancer Control 8th edition) (**Fig. 5A** and **5B**).

Postoperatively, the patient experienced biliary leakage, which was resolved with conservative management. He was discharged on POD 45. The patient received adjuvant chemotherapy with S-1 and was followed up on an outpatient basis.

At 20 months post-surgery, bilateral lung metastases were detected. At 22 months, the left lung metastasis was surgically resected and confirmed as a metastatic tumor from the initial IPNB on histological examination. GCS therapy was resumed, and no new lesions appeared. At 33 months post-hepatectomy, the right lung metastasis was resected and also confirmed as IPNB metastasis.

As of 38 months after liver resection, the patient remains tumor-free.

## DISCUSSION

Tumor thrombosis is most commonly observed in hepatocellular carcinoma, but can also occur in bile duct cancer. In our case, tumor thrombosis was suspected on imaging, but pathology showed only organized thrombus, and no tumor cells were observed within the vein. The exact cause of RHV thrombosis in this case remains uncertain. However, given the absence of tumor invasion into the vein on imaging or pathology, and the fact that thrombus formation occurred shortly after PVE, we speculate that local hemodynamic changes or hypercoagulability associated with the malignant tumor may have contributed to the thrombus formation. Although a tumor thrombus was initially suspected, pathological examination did not support this diagnosis.

In this case, thrombosis developed after preoperative PVE. Chemotherapy was administered first, considering both the risk of postoperative liver failure and the potential for curative treatment. Previous reports have suggested that the presence or absence of intrahepatic venous shunts, which drain areas of venous reflux, affects postoperative liver regeneration in hepatectomy with hepatic vein resection.^6)^ These shunts may be identified via preoperative 3D CT or venography, though reported detection rates vary widely—from 17% to 83%.^6)^ While intrahepatic venous shunts are not rare, their frequency and morphology vary significantly depending on individual anatomy and hemodynamic adaptation. Some reports indicate that the development of collateral venous pathways is difficult to assess preoperatively using imaging alone.^7)^ Nagino et al. reported performing simultaneous left portal vein and RHV embolization, which increased blood flow through the existing IRHV and resulted in hypertrophy of the right posterior segment, enabling left trisectionectomy while preserving the IRHV.^2)^ In a previous report,^2)^ CT performed 14 days post-embolization demonstrated sufficient portal venous perfusion in the right posterior segment, with no difference in contrast enhancement between ventral and dorsal regions—suggesting the formation of collateral vessels between the RHV and IRHV.

**Fig. 6:**
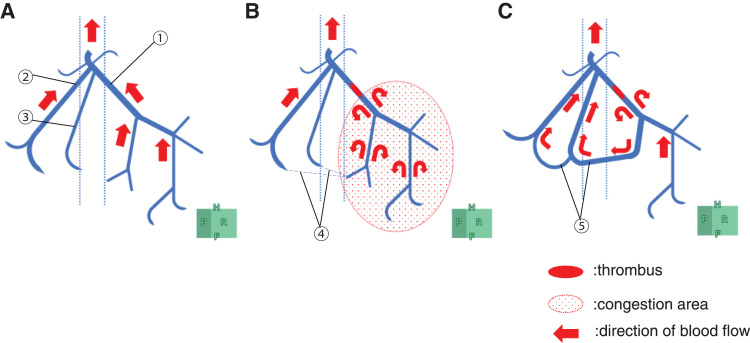
Schematic illustration of the RHV thrombus and collateral development. (**A**) Before thrombosis formation, (**B**) immediately after thrombus formation, and (**C**) after shunt formation. Thrombosis in the RHV (①) causes congestion in part of the posterior region. Immediately after thrombosis, no obvious shunt flow was observed between the occluded RHV and the branches of V7 (②, ③). However, a peripheral venous shunt (④) gradually formed, and ultimately, blood from the congested area of the occluded thrombus returned to the inferior vena cava via the developed venous shunt (⑤), alleviating the congestion. RHV, right hepatic vein

In our case, the RHV obstruction was due to thrombus. The IRHV was not recognized, and initially, the drainage vein of the RHV could not be identified. However, during chemotherapy, collateral flow developed between the peripheral branches of the RHV. Morel et al. reported that in 32 cases of MHV or RHV resection, MHV–RHV shunt vessels were detected preoperatively on CT in 20 cases and were confirmed postoperatively.^8)^ Other studies have identified shunts between small veins in the RHV drainage area and the MHV, which enabled bisegmentectomy of segments 7 and 8 with RHV resection even in the absence of a developed IRHV.^9)^ We suspect that a small venous anastomosis between the RHV and its peripheral branches existed initially but became dilated and functionally significant owing to thrombotic obstruction and associated intrahepatic pressure changes (**Fig. 6A**–**6C**).

With ongoing chemotherapy, the thrombus regressed, and both the FLR and fFRLV met our department’s criteria for liver resection, leading to a successful left trisectionectomy with bile duct resection. This case highlights the importance of continuous imaging assessment in cases initially considered unresectable owing to vascular complications. If dynamic changes in blood flow—such as shunt formation—can be confirmed during chemotherapy, conversion to resectability may become possible.

This case provides valuable clinical insight into how strategic re-evaluation, including chemotherapy and repeated imaging, can enable resection in patients with biliary tract tumors initially deemed unresectable. Although IPNB is considered an early-stage malignancy with slow growth,^10)^ it carries the risk of progression to invasive carcinoma. Therefore, careful and short-term follow-up is strongly recommended even after curative resection.^11)^ Follow-up should include both assessment for recurrence and evaluation of intrahepatic blood flow.

## CONCLUSIONS

We experienced a case of IPNB with RHV thrombosis, in which collateral shunt formation during chemotherapy enabled successful left trisectionectomy with bile duct resection. This case underscores the importance of dynamic imaging assessment in reevaluating resectability in initially unresectable patients.
